# Water Flow and Light Availability Influence on Intracellular Geosmin Production in River Biofilms

**DOI:** 10.3389/fmicb.2019.03002

**Published:** 2020-01-14

**Authors:** Carmen Espinosa, Meritxell Abril, Helena Guasch, Núria Pou, Lorenzo Proia, Marta Ricart, Marc Ordeix, Laia Llenas

**Affiliations:** ^1^BETA Technological Center, University of Vic – Central University of Catalonia, Vic, Spain; ^2^Center for the Study of Mediterranean Rivers, University of Vic – Central University of Catalonia, Manlleu, Spain; ^3^Research Group on Ecology of Inland Waters, Institute of Aquatic Ecology, University of Girona, Girona, Spain; ^4^Centre d’Estudis Avançats de Blanes, Consejo Superior de Investigaciones Científicas, Blanes, Spain

**Keywords:** geosmin, water flow, light availability, biofilm, mesocosms

## Abstract

Hydro-morphological alterations in water bodies caused by climate change and human activities affects the ecosystem functioning and generate important water quality problems. Some of these alterations can generate an increase in cyanobacterial blooms, which are associated with the appearance of bad taste and odorous compounds such as geosmin. The factors that trigger their production are still unclear, and this inability to predict geosmin episodes provokes economic problems for water supply companies. This study aims to evaluate the effects of water flow and light availability on biofilm development and intracellular geosmin formation. A mesocosm experiment was performed between February–April, 2019. The mesocosms were a set of 10 outdoor 3 m long flumes, with a continuous water supply from the Ter river (Catalonia, NE Spain). Two light intensities were established: natural light and light reduced to 80%, combined with five gradual water flows from 0.09 to 1.10 L/s. Water samples were taken to analyze nutrients, and biofilm samples, to analyze geosmin concentration, chlorophyll *a* and the community. Geosmin in biofilm was detected in those treatments in which *Oscillatoria* sp. appeared. The concentration of intracellular geosmin was higher at lower water flows (0.09 and 0.18 L/s), and the highest (2.12 mg/g) was found in the flume with the lowest water flow (0.09 L/s) and irradiation (20%). This flume was the one that presented a greater concentration of *Oscillatoria* sp. (21% of the community). It stands out that, when geosmin in biofilm was found, the dissolved inorganic nitrogen and soluble reactive phosphorus ratio decreased, from an average of 417:1 to 14:1. This was mainly due to an increase in inorganic phosphorus concentration generated by a change in the nutrient uptake capacity of the community’s biofilm. The results obtained in this study indicated the potential implications for stream ecosystem management to control geosmin appearance. Likewise, they could be used as an early warning system, establishing that in times of drought, which lead to a general decrease in river water flow, the situation could be optimal for the appearance and development of geosmin producing cyanobacteria in low-flow areas near the river banks.

## Introduction

Due to several natural and anthropogenic factors such as nutrient enrichment, temperature increase and pollutant loading, cyanobacterial blooms are increasing worldwide (Vilalta, 2004; Vahtera et al., 2007; Winter et al., 2011). These cyanobacterial blooms are usually responsible for the appearance of non-desirable metabolites, toxics in many cases (Lee et al., 2017). Moreover, these blooms are associated with the release in waterbodies of Taste and Odor compounds (T&Os) which are becoming a problem in waterbodies management. Although no direct health risk has been associated with the T&Os (Tung et al., 2008), they influence the aesthetic water quality and consumer trust in drinking water companies (Ding et al., 2014). These compounds are perceived from humans at very low concentration (Smith et al., 2009), resulting in consumer complaints, especially during the outbreak period of producers blooms. These metabolites can also negatively affect the marketability of aquaculture products (Schrader and Rimando, 2003). Odor episodes in drinking water have become an universal water issue reported worldwide (Tung et al., 2008). Exact causes and conditions under which cyanobacteria begin to produce these compounds have not been completely defined, since companies have prioritized the definition of strategies to treat and eliminate these compounds instead of evaluating the factors associated with their production in the ecosystem (Jüttner and Watson, 2007).

Diverse organisms have been identified as T&Os producers (Li et al., 2012; Jørgensen et al., 2016), including eukaryotes, such as fungi and amoeba, with photoautotrophic cyanobacteria and filamentous heterotrophic bacteria being the main producers (Olsen et al., 2016). Among the T&Os produced by microorganisms, geosmin is one of the most commons and cyanobacteria are considered the major geosmin producing-organisms in freshwater environments. The majority of the cyanobacteria known as geosmin producers are benthic or epiphytic (70%), whereas the remainder are planktonic (Jüttner and Watson, 2007). Several geosmin-producing cyanobacteria have been identified, being *Oscillatoria* sp., *Lynghya* sp., *Symploca* sp., and *Anabaena* sp. the most commonly found in freshwater ecosystems (Smith et al., 2009).

Geosmin is a bicyclic terpenoid, responsible for earthy and musty odor in drinking water (Watson et al., 2016). Geosmin is a volatile compound that is stored inside the cell in gas vacuoles and it is released in response to cellular death and/or biomass decomposition (Lee et al., 2017). Thus, geosmin can appear in surface waters of freshwater ecosystems exploited for drinking purposes such as lakes, reservoirs, and rivers. Although numerous advances have been made to understand the mechanisms of geosmin production at cellular level, its biological function has not been elucidated yet. One of our hypotheses is that geosmin is a residual metabolite potentially providing energetic benefit to the producing organism. In particular, considering that the energy requirement for chlorophyll *a* (Chl *a*) production is higher than geosmin, it has been described that when the organisms do not need high Chl *a* concentration, eventually they start to produce geosmin.

Different environmental factors (i.e., temperature) may influence the microbial production of geosmin (Lee et al., 2017), however, these isolated factors can not explain the differences in geosmin concentration observed in freshwater under natural conditions (Jüttner and Watson, 2007). Most cyanobacteria species are known to have optimum temperature growth conditions (>20°C), but many filamentous cyanobacteria have the ability to adapt to lower temperature by controlling its pigments levels (Tang et al., 1997). Light is also known to affect the production of some volatile organic compounds (VOCs) and the growth of algal species (Lee et al., 2017; Alghanmi et al., 2018). It has been shown that high light availability can inhibit the formation of gaseous vacuoles in certain cyanobacteria (Blevins et al., 1995; Li et al., 2012) and several studies have pointed light as a key factor for geosmin production (Saadoun et al., 2001; Wang and Li, 2015; Lee et al., 2017). Water flow may also have a certain effect on microbial production of T&Os, indeed, elevated geosmin concentrations have been reported in proximity to dams of several rivers. In these areas, the flow is significantly reduced, leaving the water stagnant (Jüttner and Watson, 2007). These two factors, light intensity and water flow, can be altered by human activity and climate change (Elosegi and Sabater, 2013).

Global climate models predict changes that will generate more frequent and intense extreme events in next decades (Acuña and Tockner, 2010), altering the natural flow regimes of rivers and streams. Specific predictions for Mediterranean climate regions indicate a strong reduction in the mean precipitation — which would occur as more intense and less frequent rainfall events—and a probable decrease in annual runoff that will lead to reduced river flows (Bangash et al., 2013; Proia et al., 2013; Schneider et al., 2013). In addition, hydrological regime of running waters can be affected by flow regulation through dams and impoundments building (Rubio-Gracia et al., 2017). Moreover, anthropogenic activities can alter river banks causing riparian forest modifications and consequentially altering the quantity and quality of light potentially reaching riverbeds (Dal Sasso et al., 2015). Variation of light and/or water flow regimes accounts for much of the variation in the physiology, growth, structure and functioning of benthic microbial communities (von Schiller et al., 2008), and therefore, they can represent indirect drivers of geosmin production and release in rivers.

The Ter river born in the middle of the Catalan Pyrenees (at 2400 m high) flows into the Mediterranean Sea. It has a 3010 km^2^ of basin surface and 208 km of longitude. The Mediterranean climate determines the rain patterns over the basin, which is characterized by maximum rainfall occurring during spring and autumn with dry and warm summers. Moreover, in its mid-section there are some anthropogenic activities such as (i) a high number of dams used to obtain hydraulic energy, (ii) livestock farming and agriculture, leading to an increase in nutrients concentration and (iii) a system of reservoirs (Sau, Susqueda, and El Pasteral) with a capacity of 375 Hm^3^, which supplies raw water for drinking purposes. All these activities are drastically affecting water flow and water quality downstream of the river (Espadaler et al., 1997; Céspedes et al., 2008). In addition, geosmin episodes in the Ter river basin have increased in the last decade of 21^*th*^ century, after the increase of urban and industrial activities in the upper part of the Ter river basin (Céspedes et al., 2008). This is a concern for drinking water treatment companies in the area, as they are not able to predict the presence of geosmin in the river and, therefore, apply the specific treatment when needed.

Although it has been described that geosmin production depends on environmental conditions, most of the studies were carried out with single specie cultures under controlled conditions (Schrader and Blevins, 2001; Parinet et al., 2010; Li et al., 2012; Suurnäkki et al., 2015; Wang and Li, 2015), and there are very few studies dealing with drivers of geosmin occurrence under natural environmental conditions in complex microbial communities. It is therefore essential to understand how the variation of multiple environmental variables can affect biofilm community structure and subsequently geosmin formation. This study was designed to explore the combined effects of light availability and water flow on biofilm structure and functioning, and its relationship with intracellular geosmin formation. The hypothesis is that geosmin formation is fostered by a decrease in water flow, since this situation is the most suitable for the growth of cyanobacteria; and by the decrease in light intensity, since high light intensity could inhibit the gas vacuole formation (Blevins et al., 1995). Thus, we predicted greater geosmin-producing cyanobacteria abundance under low water flow conditions, that combined with low light intensity, would promote the geosmin formation in biofilm. To test this, an experiment was carried out with outdoor mesocosms that received water from the Ter river (Catalonia, NE Spain).

## Materials and Methods

### Experimental Design and Sampling Procedure

**FIGURE 1:**
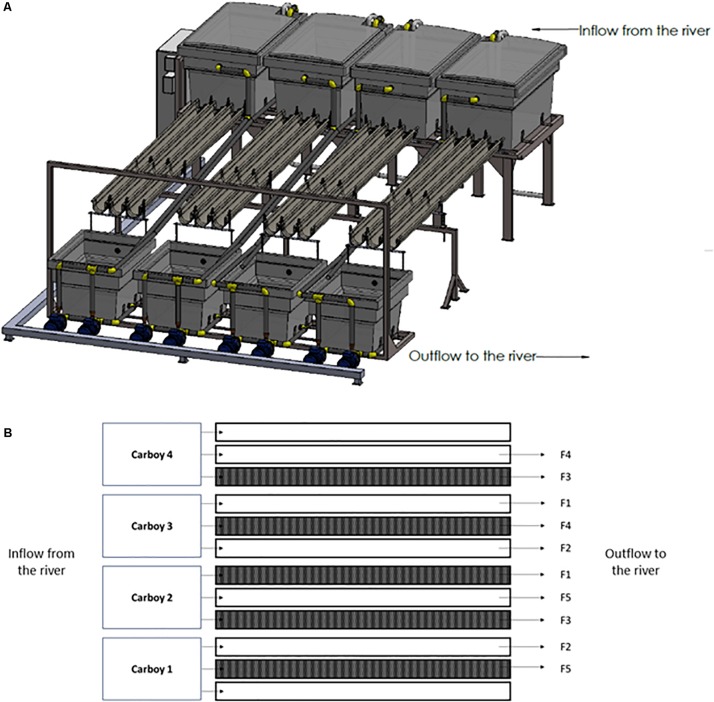
Experimental rivers representation. **(A)** Three-dimensional representation, **(B)** scheme of the experimental design carried out where F1 = 0.09 L/s; F2 = 0.18 L/s; F3 = 0.36 L/s; F4 = 0.72 L/s; and F5 = 1.10 L/s. The dark represented flumes are those covered during the experiment for the Low Light treatment.

In this experiment, 10 outdoor experimental flumes (3.5 m long × 12 cm wide × 8 cm high), which receive water directly from an industrial channel of the mid-section Ter river in Manlleu (NE Catalonia, Spain), have been used (Figure 1A). The experiment was conducted from late March till April in 2018, since it is known that in the Ter river this is the period in which a greater production of geosmin is expected.

The river water was pumped from the river channel by four submerged pumps (drainage pump, VXV 1100AS) into four 1000 L carboys, each of them feeding three experimental flumes that discharge into the same channel downstream of water abstraction.

The two parameters manipulated in this study were light intensity and water flow. Particularly, two light and five water flow conditions were set for the experiment. Five flumes were left under natural light conditions (High Light treatment, HL = 515 ± 57 μmol photons/m^2^⋅s) whereas the rest were covered with a greenhouse net to achieve an 80% reduction of the light reaching the flumes (Low light treatment, LL = 107 ± 38 μmol photons/m^2^⋅s). This light availability reduction was chosen according to the light availability common for streams in a well-develop riparian forest (Hill et al., 2009; Bowes et al., 2012). Light intensity was monitored continuously during the experiment using HOBO Data Loggers (Hobo^®^ Pendant, Onset Computer, Corp., Bourne, MA, United States). By manipulating the independent tap of each flume, five levels of water flow were set: F1 = 0.09 L/s; F2 = 0.18 L/s; F3 = 0.36 L/s; F4 = 0.72 L/s; and F5 = 1.10 L/s, in both HL and LL flumes. Water flow was regularly quantified by measuring the volume of water per minute outflowing from each flume. Both, light and water flow conditions, were randomly distributed across the experimental flumes (Figure 1B).

Thirty river cobbles, previously scraped and autoclaved, were placed in each flume to allow biofilm colonization using inflowing river water as natural inoculum. Biofilm development and physicochemical parameters were monitored once per week throughout the experiment and a sampling was carried out at the end of the experiment, after 41 days, to evaluate the overall response of the biofilms to the treatments.

Physicochemical parameters were measured *in situ* with specific probes: temperature, dissolved oxygen concentration, and oxygen saturation (YSI professional plus, YSI Incorporated, United States), pH (XS pH7 + DHS) and conductivity (XS COND 7+).

At the inlet and outflow of each flume, three water samples were taken per sampling day. Samples were filtered through 0.45 μm cellulose filters before the analysis of soluble reactive phosphorus (SRP), N-NH${}_{4}^{+}$, N-NO${}_{2}^{-}$, and N-NO${}_{3}^{-}$. All samples were stored at −20°C until analysis.

The last day of the experiment, three cobbles from each flume were randomly collected and scraped in 30 ml of water from the same flume to obtain a biofilm suspension. Aliquots of these suspensions were used to analyze geosmin concentration, Chl *a*, ash free dry mass (AFDM), algal taxonomic composition and photosynthetic efficiency. The photosynthetic efficiency was measured *in situ* with an amplitude modulated fluorimetry (Mini-PAM fluorometer Walz, Effeltrich, Germany). Samples were stored frozen at −20°C until analyses, except aliquots for geosmin concentration that were frozen at −80°C and taxonomic samples, which were fixed with formalin (2%) and stored at 4°C.

### Biofilm Samples

#### Chlorophyll *a*

Algal biomass was estimated from the analysis of Chl *a* concentration in biofilms. This analysis was performed using the protocol described by Artigas et al. (2015). Chlorophyll *a* was extracted from the sample with 90% acetone over a period of 12 h in the dark at 4°C. Acetone extracts were filtered through 0.7 μm glass fibber filters (GF/F filters, Whatman), and Chl *a* concentration was determined spectrophotometrically (NanoPhotometer^TM^ P-360) following the method described by Jeffrey and Humphrey (1975). Results are given as μg Chl *a* per stone surface area (cm^2^).

#### Ash Free Dry Mass

Total biofilm biomass was measured as AFDM using the protocol defined by Proia et al. (2016). The aliquots of biofilm suspension were filtered through pre-combusted (4 h at 500°C, Carbolite muffle ELF 11/14B) and pre-weighed 47-mm GF/F Whatman glass-fiber filters (0.7 μm pore size), then dried for 72 h at 60°C (Forced air oven, MEMMERT IFE500) in order to calculate the dry matter (DM). Afterward, samples were combusted at 500°C (Carbolite muffle ELF 11/14B) for 4 h, and then weighed again. The AFDM was calculated by the differences in filter mass before and after drying (72 h at 60°C) and after combustion (4 h at 500°C). Results are expressed as g organic matter (OM)/cm^2^. The Margalef Index (MI) was calculated as the quotient between the carotenoid/Chl *a* ratio (Eq. 1):

(1) M⁢I=A⁢b⁢s⁢.430⁢n⁢m/A⁢b⁢s⁢.665⁢n⁢m

This index is based on the fact that Chl *a* is the pigment that is synthesized and degraded more quickly, while other pigments accumulate and are more resistant to degradation than Chl *a*. Thus, all populations that have low productivity, are characterized by lower concentrations of pigments and, therefore, the relationship of Chl *a* with the rest of the pigments is lower or higher depending on the age of the populations. Therefore, with this index, low values are obtained in population that grow rapidly and increase when the population is more mature and with a low renewal rate (Margalef, 1960).

The Autotrophy Index (AI) (Eq. 2) was also calculated as:

(2) A⁢I=A⁢F⁢D⁢M/C⁢h⁢l⁢a

This index represents the theoretical limit of 200, therefore, values above 200 indicate a predominance of heterotrophs, and lower values of autotrophs (Weber, 1973).

#### Biofilm Community

Cell biovolume was obtained following the procedure described in Hillebrand et al. (1999). To calculate the biovolume, the width and length of at least 10 individuals, randomly selected, were measured for each genus. The optical microscope (Nikon Eclipse 600W) using phase-contrast and Nomarski differential interference contrast optics at a magnification of 400 increments was used, counting and measuring the cells of a minimum of 6 fields of vision per sample. The values were transformed as described in Ricart et al. (2009), using the surface of the sample, the dilutions needed and the number of cells counted, and were expressed as μm^3^/cm^2^.

#### Geosmin

For the analysis of geosmin in biofilm, the protocol established by Wang and Li (2015) was used. Samples were frozen and thawed five times and subjected to sonication to facilitate cell breakage and geosmin release. Headspace solid phase micro-extraction was performed according to Klausen et al. (2005). Briefly, 10 mL of the sample was decanted in a 100 mL opaque reaction vial, that contained 10 g of NaCl and 40 mL of sterile dH_2_O, to obtain 50 mL of total volume, and a magnetic stir bar. A 65 μm PDMS/DVB fibber was injected into the headspace of the sample vial, and the vial was placed on a magnetic stirrer inside an oven at 60°C for 25 min to extract geosmin from the samples. Geosmin characterization was performed using a GC/MS instrument (ISQ – TRACE GC ULTRA). Geosmin was separated using a capillary column (Sigma Aldrich SPB^®^ -5 Capillary GC Column) measuring 30 m x 0.25 mm with a film thickness of 0.25 μm. Carrier gas was helium at a flow rate of 1 mL/min). Geosmin was desorbed by exposing the fibber in the GC injection port for 6 min at 250°C. A 0.75 mm internal diameter glass liner was used, and the injection port was in splitless mode. The temperature program was isothermal for 3 min at 50°C, raised to 250°C at a rate of 30°C/min and, finally, to 300°C at a rate of 10°C/min. The transfer line to the mass spectrometer and the ion source were 250 and 200°C respectively, the scan was in SIM mode being the scanned fragments 111 and 112 m/z. When geosmin was detected but its concentration was below the quantification limit (2.5 ng/L), half of the quantification limit (1.25 ng/L) was assumed as concentration (Bonet et al., 2013).

### Water Samples

#### Nutrients

Soluble reactive phosphorus concentration in water samples was measured using the protocol described by Murphy and Riley (1962) and ammonium concentration was analyzed following the protocol described in Reardon et al. (1966). Nitrate and nitrite concentration were determined following the methods described in Rand et al. (1976). In all of these protocols, the absorbance was measured with the spectrophotometer NanoPhotometer^TM^ P-360.

#### Nutrients Uptake Capacity

The nutrients uptake capacity of biofilms was estimated considering the difference in nutrients concentrations between the input and the outflow of each flume taking into account the area colonized by biofilms (Proia et al., 2017). The uptake capacity (U, mg/m^2^) (Eq. 3) was calculated as:

(3) $$U=\frac{\left(C_{i}\cdot V_{i}\right)-\left(C_{f}\cdot V_{f}\right)}{A}$$

where C_*i*_ = the initial nutrient concentration; V_*i*_ = the initial volume; C_*f*_ = the final nutrient concentration; V_*f*_ = the final volume; and A = the area colonized by the biofilm.

### Data Treatment

Physicochemical and biological data were evaluated using an analysis of covariance (ANCOVA) in SPSS Statistics version 21, with water flow being the independent variable and light availability the covariate. The distribution of the phototrophic community of the biofilm according to the treatments was evaluated using a principal component analysis (PCA), and Pearson correlation coefficient test were performed to explore the relationship between intracellular geosmin concentration and the biological and the physicochemical variables. Statistical significance was set at *p* < 0.05 for all test performed.

## Results

### Physicochemical Parameters

The experimental conditions of the carboys and flumes remained stable throughout the experiment, with low water temperature, high oxygen water levels, slight alkaline pH, and medium mineralization (Table 1). There were not significant statistically differences among the carboys along the experiment (ANCOVA, *p* = 0.781). Nutrient concentrations in the carboys varied throughout the time as a consequence of the variations naturally occurring in Ter river, which supplied the water to the mesocosms. This variation was the same for all the flumes.

**TABLE 1:** Mean value and standard deviation of the physicochemical variables evaluated in the flumes throughout the experiment (*n* = 84), under low light (LL) and high light (HL) conditions for the different flow treatments (F): pH, water temperature (°C), electrical conductivity (EC) (μS/cm), dissolved oxygen (DO) (mg/L), oxygen saturation (%), water flow (L/s), light irradiance (μmol photons/m^2^⋅s), ammonium (μg N-NH${}_{4}^{+}$ /L), nitrite (μg N-NO${}_{2}^{+}$ /L), nitrate (mg N-N0_3_^+^/L) and phosphate concentration (μg P-PO${}_{4}^{3-}$ ^/^L).

	**Low light (LL)**					**High light (HL)**				
	**F1**	**F2**	**F3**	**F4**	**F5**	**F1**	**F2**	**F3**	**F4**	**F5**
pH	8.3 ± 0.3	8.4 ± 0.2	8.4 ± 0.2	8.4 ± 0.2	8.4 ± 0.2	8.4 ± 0.2	8.3 ± 0.3	8.4 ± 0.2	8.4 ± 0.2	8.4 ± 0.2
Water temperature (°C)	10 ± 3	10 ± 3	9 ± 3	10 ± 3	10 ± 3	10 ± 3	10 ± 3	10 ± 3	10 ± 3	10 ± 3
EC (μS/cm)	332 ± 58	339 ± 50	337 ± 55	335 ± 54	333 ± 56	333 ± 50	336 ± 52	336 ± 53	334 ± 59	340 ± 55
DO (mg/L)	10.8 ± 1.5	11.4 ± 0.9	11.3 ± 0.8	11.3 ± 0.9	11.3 ± 0.9	11.1 ± 1.1	11.3 ± 0.8	11.4 ± 0.8	11.3 ± 0.7	11.4 ± 0.7
Saturation (%)	95 ± 13	100 ± 6	99 ± 6	99 ± 8	99 ± 6	98 ± 9	100 ± 6	100 ± 6	99 ± 5	99 ± 7
Water flow (L/s)	0.09 ± 0.00	0.18 ± 0.00	0.31 ± 0.02	0.61 ± 0.04	1.20 ± 0.09	0.09 ± 0.01	0.17 ± 0.01	0.33 ± 0.03	0.60 ± 0.07	1.14 ± 0.02
Light irradiance (μmol photons/m^2^⋅s)	110 ± 15	118 ± 13	114 ± 16	109 ± 21	112 ± 15	509 ± 32	516 ± 18	519 ± 22	512 ± 17	511 ± 28
N-NH${}_{4}^{+}$ (μg/L)	74 ± 62	74 ± 62	75 ± 60	78 ± 70	76 ± 59	78 ± 70	73 ± 59	78 ± 70	74 ± 62	75 ± 60
N-NO${}_{2}^{-}$ (μg/L)	8 ± 5	6 ± 4	9 ± 7	7 ± 4	7 ± 6	8 ± 7	6 ± 4	6 ± 4	6 ± 3	7 ± 5
N-NO${}_{3}^{-}$ (mg/L)	0.9 ± 0.3	0.9 ± 0.3	0.9 ± 0.3	0.8 ± 0.3	0.9 ± 0.3	0.9 ± 0.2	0.9 ± 0.2	0.8 ± 0.2	0.9 ± 0.2	0.9 ± 0.3
P-PO${}_{4}^{3-}$ (μg/L)	19 ± 6	10 ± 2	14 ± 4	13 ± 5	10 ± 3	12 ± 4	12 ± 2	13 ± 6	12 ± 5	14 ± 2

### Biofilm Attributes

#### Structural Parameters

**FIGURE 2:**
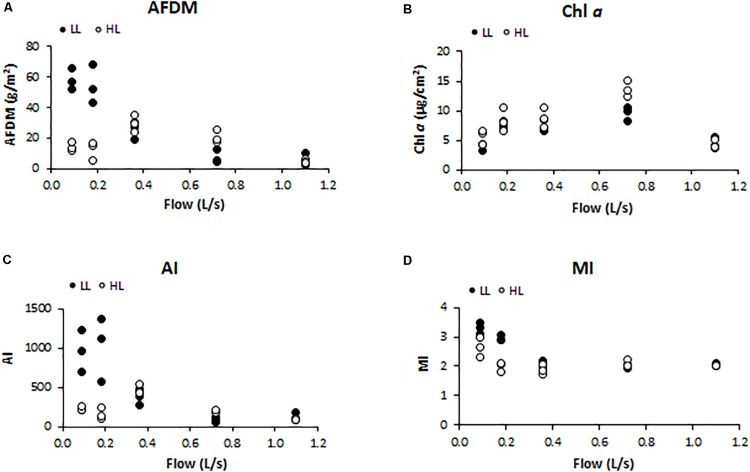
Results obtained in the different treatments for the different structural biofilm descriptors measured at the end of the experiment (*t* = 41 days): **(A)** ash free dry mass (AFDM) (g/m^2^), **(B)** Chl *a* (μg/cm^2^), **(C)** Autotrophic Index (AI) (AFDM/Chl *a*), and **(D)** Margalef Index (MI) (Abs. 430 nm/Abs. 665 nm).

Biofilm biomass measured as AFDM (g/cm^2^) showed a different pattern as response to light availability (ANCOVA, *F* = 4.560, *p* < 0.05) (Figure 2A). In particular, biofilms developed under lower flow and light conditions (F1LL, F2LL) showed significantly higher values of AFDM (60 ± 8and 56 ± 7 g/m^2^, respectively) with respect to the biofilms from higher flow and light treatments (Figure 2A). The Chl *a* (μg/cm^2^) did not present differences depending on the light (ANCOVA, *F* = 1.503, *p* = 0.230) (Figure 2B). The lowest values were found at the extreme water flows, F1 and F5, both with a Chl *a* concentration around 5 ± 1 μg/cm^2^. The Autotrophic Index (AI) was significantly affected by light (ANCOVA, *F* = 5.351, *p* < 0.05) (Figure 2C), with the flumes with low flow and low light (F1LL and F2LL) being the ones with the highest values, 837 ± 150 for F1LL and 976 ± 255 for F2LL. Similar results were observed for MI (ANCOVA, *F* = 4.528, *p* < 0.05) (Figure 2D), with biofilms developed in flumes with lower flows (F1 and F2) showing higher values than the others. In particular, under low light conditions biofilms showed greater values of MI, being F1LL the highest (3.2 ± 0.2).

In LL conditions, water flow was negatively correlated with AFDM (Pearson’s correlation, *r* = −0.829, *p* < 0.001), AI (*r* = −0.735, *p* < 0.01), and MI (*r* = −0.718, *p* < 0.01); in HL conditions, no correlations were found between these structural parameters and water flow.

**FIGURE 3:**
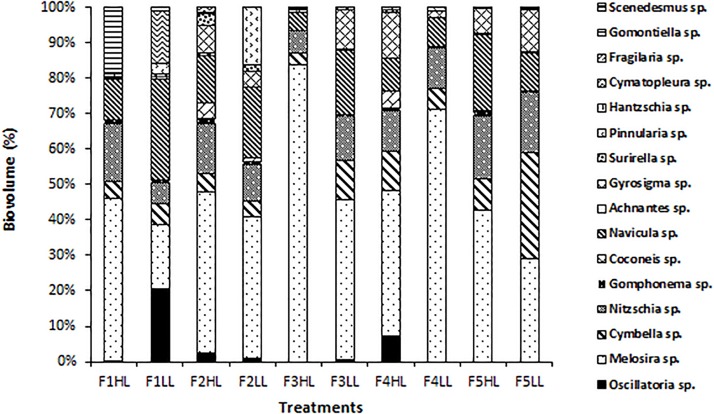
Biovolume (in%) of algae, diatoms and cyanobacteria present in the biofilm of each treatment, from the lowest flow (F1) to the highest (F5), under conditions of natural light (HL) and reduced (LL).

**FIGURE 4:**
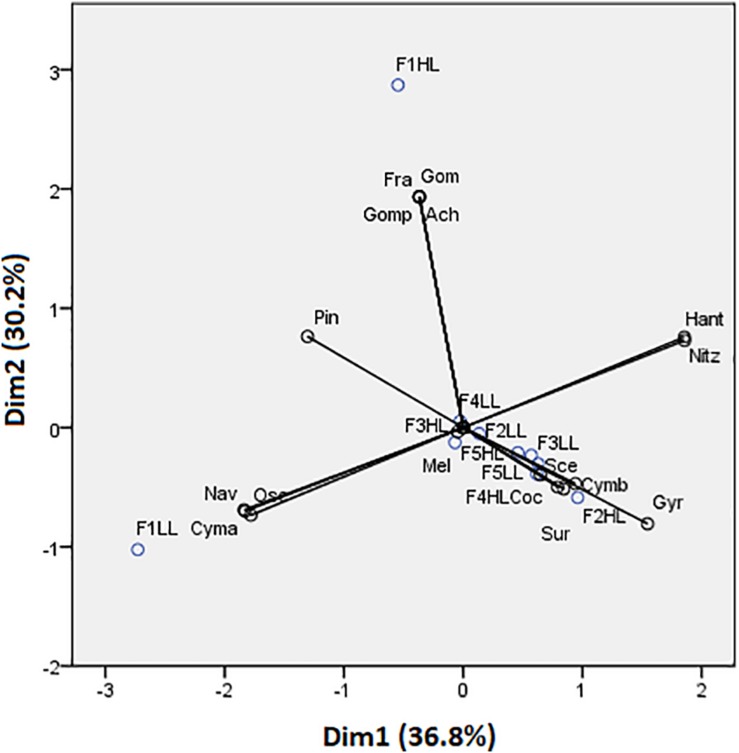
Principal component analysis (PCA) showing flumes distribution based on biofilm species. Axes 1 and 2 combined explain 66.98% of the variance. Cyanobacteria: Osc = *Oscillatoria* sp. and Gom = *Gomontiella* sp.; diatoms: Mel = *Melosira* sp.; Cmb = *Cymbella* sp.; Nit = *Nitzschia* sp.; Gomp = *Gomphonema* sp.; Coc = *Cocconeis* sp.; Nav = *Navicula* sp.; Ach = *Achnantes* sp.; Gyr = *Gyrosigma* sp.; Sur = *Surirella* sp.; Pin = *Pinnularia* sp.; Han = *Hantzschia* sp.; Cym = *Cymatopleura* sp.; Fra = *Fragilaria* sp.; and green algae: Sce = *Scenedesmus* sp.

Phototrophic community structure was different among flumes (Figure 3), particularly, changes were detected on the relative abundances (expressed as biovolume) of *Oscillatoria* sp. (ANCOVA, *F* = 0.175, *p* < 0.001), *Melosira* sp. (ANCOVA, *F* = 1.268, *p* < 0.05), and *Pinnularia* sp. (ANCOVA, *F* = 4.358, *p* < 0.05). Precisely, the PCA evidenced that the biofilms developed under lower flow conditions (F1) were different in terms of relative abundances of the taxa (Figure 4). Light availability influenced the community structure. Indeed, F1LL biofilms were separated from the rest mainly driven by the higher relative abundance of *Oscillatoria* sp., among others (Figures 3, 4). On other hand, the biofilm in F1HL flume was also differentiated from the rest by the presence of *Fragilaria* sp., *Gomontiella* sp., and *Achnantes* sp. The relative abundance of the genera *Oscillatoria* sp. was affected by the interaction of light and water flow, being dominant in biofilms developed under lower flow and low light availability (F1LL).

#### Functional Parameters

##### Photosynthetic capacity

The biofilm photosynthetic capacity (expressed as Ymax) showed significant differences depending on light (ANCOVA, *F* = 1.607, *p* < 0.05), being slightly superior under LL conditions. There was a positive correlation between the water flow and Ymax (*p* < 0.05), where the biofilms growing under lower flow conditions (F1) exhibited the lowest values (0.347 ± 0.085 for F1LL and 0.278 ± 0.005 for F1HL).

##### Nutrient uptake capacity

**FIGURE 5:**
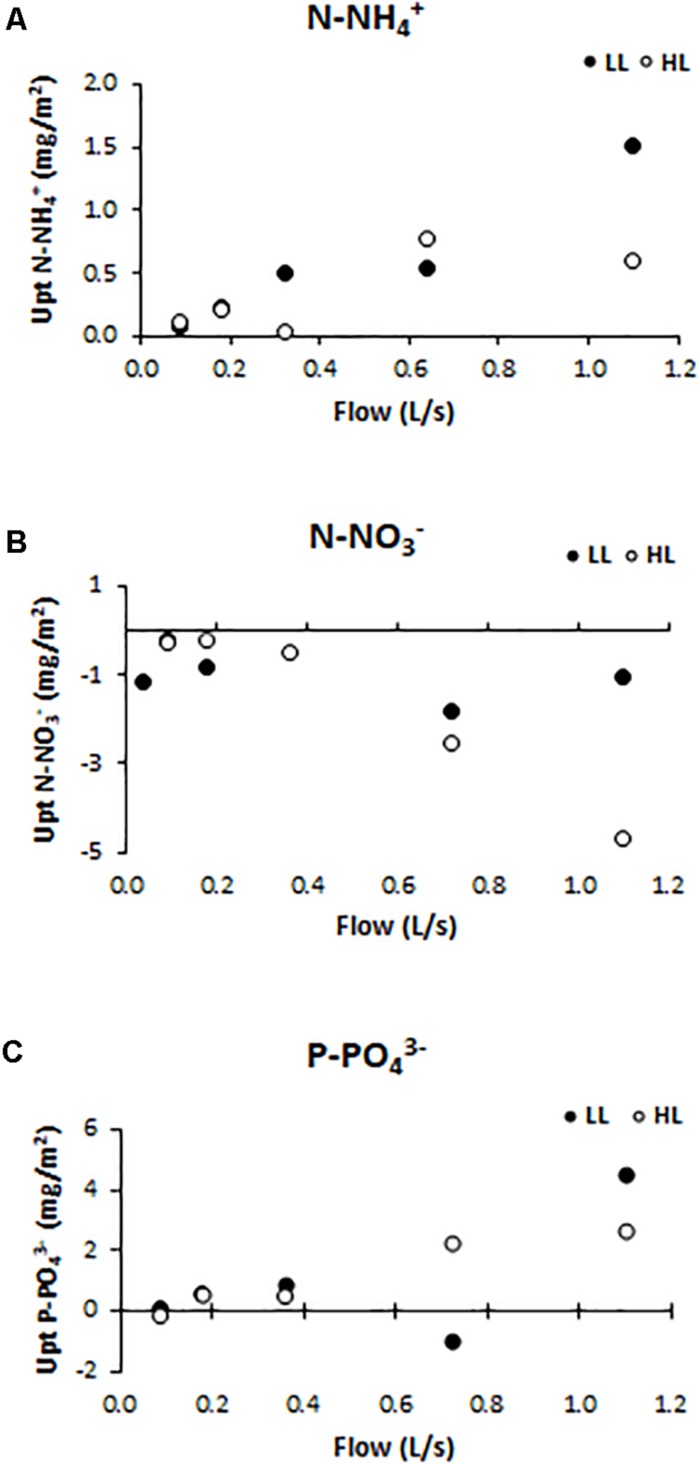
Uptake/release capacity values for different treatments: **(A)** ammonium (mg N-NH${}_{4}^{+}$ /m^2^), **(B)** nitrate (mg N-NO${}_{3}^{-}$ /m^2^), and **(C)** phosphate (mg P-PO${}_{4}^{3-}$ /m^2^).

Nutrient uptake (or release) capacity of ammonium (Figure 5A), nitrate (Figure 5B), and phosphate (Figure 5C), were significantly affected by light conditions (ANCOVA, *F* = 12.153, *p* < 0.001 for the ammonium; *F* = 416.361, *p* < 0.001 for the nitrate, and *F* = 119.28, *p* < 0.001 for the phosphate). The ammonium uptake capacity tended to increase at higher flows, reaching, under LL conditions, the values of 1.05 mg N-NH${}_{4}^{+}$/m^2^ (F5LL). Nitrate was normally not consumed but released by biofilms along the flumes, as a probable consequence of nitrification activity within the communities. The nitrate release capacity showed the opposite trend with respect to ammonium uptake capacity pattern. Indeed, the highest release value (4.68 mg N-NO${}_{3}^{-}$/m^2^) was found in flumes with higher flow and light availability (F5HL). Phosphate uptake capacity had the same pattern as ammonium uptake capacity; being higher in biofilms grown under higher flow and lower light availability conditions when the uptake values were higher (4.50 mg P-PO${}_{4}^{3-}$/m^2^). If the DIN:SRP ratio is calculated; being DIN the sum of the dissolved inorganic nitrogen, it is observed that the differences in nutrient uptake dynamics were reflected in the water DIN:SRP ratio which showed differences depending on the treatment, being lower at low flows (14:1) and increasing notably in the highest flow flumes (417:1).

### Geosmin Concentration in Biofilm

**FIGURE 6:**
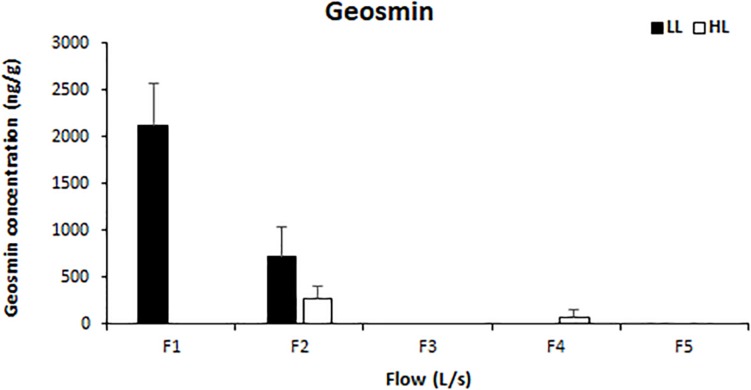
Mean values and standard deviation for intracellular geosmin concentration (ng/g) in biofilm of each treatment, from the lowest flow (F1) to the highest (F5), under conditions of natural light (HL) and reduced (LL), at the end of the experiment (*t* = 41 days).

Light availability had a significant effect on the concentration of intracellular geosmin (ANCOVA, *F* = 72.641, *p* < 0.001), which also presented a negative correlation with water flow (Pearson’s correlation, *r* = 0.918, *p* < 0.01). Geosmin was detected above the quantification limit in the biofilms of four treatments: F1LL, F2LL, F2HL, and F4HL. The highest concentrations were measured in biofilm developed under lower flow and light conditions reaching 2118 ± 460 ng/g at F1LL and 710 ± 326 ng/g at F2LL, respectively (Figure 6).

The geosmin concentration in biofilm correlated positively with different variables: *Oscillatoria* sp. biovolume (Pearson’s correlation, *r* = 0.776; *p* < 0.01), *Oscillatoria* sp. relative abundance (Pearson’s correlation, *r* = 0.924, *p* < 0.0001), AFDM (Pearson’s correlation, *r* = 0.817, *p* < 0.01), MI (Pearson’s correlation, *r* = 0.857, *p* < 0.01), and AI (Pearson’s correlation, *r* = 0.769, *p* < 0.01). Regarding the physicochemical variables, geosmin concentration in biofilm correlated positively with phosphate concentration (Pearson’s correlation, *r* = 0.963, *p* < 0.001), and temperature (Pearson’s correlation, *r* = 0.761, *p* < 0.05), and negatively with nitrite concentration (Pearson’s correlation, *r* = −0.887, *p* = 0.001).

## Discussion

Light intensity and water flow generated differences in the biofilm structure and functioning. At functional level, low light conditions caused an increase in the photosynthetic capacity (Ymax) of the biofilm, as typically observed in biofilm communities developed under this situation (Villeneuve et al., 2009; Corcoll et al., 2012). Riegman et al. (1985) also found out this pattern for the *Oscillatoria agardhii* strain under laboratory conditions. Water flow also affected the photosynthetic capacity of the biofilm community, which was reduced at the lowest flow. This could be explained by the highest presence of *Oscillatoria* in this treatment, since some studies have found that cyanobacteria usually present low photosynthetic values compared with the rest of the algal community (Kirst et al., 2014; Luimstra et al., 2018).

Regarding nutrients uptake, the biofilm developed under LL conditions had a greater ammonium and phosphate uptake capacity, whereas the biofilm developed under HL conditions showed increased nitrate release capacities suggesting greater nitrification activity. This would be in contrast with the inhibitory effect of light on nitrifying organisms reported both in cultures and natural biofilm communities (French et al., 2012; Merbt et al., 2017). However, these studies did not consider the role of flow conditions that could influence the nutrients dynamics by modifying the interactions between dissolved molecules and benthic biota or also generating differences in the diffusion capacity (Battin et al., 2016). Overall, in this study, the ability to consume or release ammonium, nitrate, and phosphate is significantly favored at higher flows. Nevertheless, it must be taken into account that it was an approximation considering the nutrients levels measured between the entrance and the outflow of the flumes, and assuming that the nutrients load variation was a consequence of biological activity (both uptake and/or release). Even so, the difference in nutrient uptake or release has been reflected in the DIN:SRP ratio, that was on average significantly lower in F1 flumes (14:1) than in F5 ones (417:1). This could be a relevant factor related with geosmin behavior in biofilms as it has been suggested that lower DIN:SRP ratios can favor the producing organism and its harvesting activity.

At the structural level, Chl *a* concentration was affected by water flow, with lower Chl *a* concentration under the most extreme flow rates (F1 and F5). The low Chl *a* concentration found at the lowest water flow could be explained by the high concentration of geosmin detected in this treatment. In fact, geosmin and Chl *a* have the same metabolic pathway and it has been described that when cyanobacteria start synthetizing geosmin the production of Chl *a* decrease or even stops (Saadoun et al., 2001; Cai et al., 2017). Related to the treatment with the highest flow, it is known that during high water velocity, biofilms are sloughed due to high shear stress, drag and abrasion, leading to low biomass (Wellnitz and Rader, 2007; Ponsatí et al., 2016).

Biofilms growing at LL conditions had higher values of AI, suggesting that there was a lower proportion of algal biomass in comparison to the total biomass (Weber, 1973). Higher registered values of AFDM and MI may be linked to the accumulation of accessory pigments and a more aged community, as described by Marcarelli et al. (2009) and Ceola et al. (2013). Light intensity represents an environmentally important factor for cyanobacterial growth (Alghanmi et al., 2018). In the LL treatment, *Oscillatoria* genus was favored within the community, strongly suggesting that low light availability in ecosystems leads to a higher presence of geosmin-producing cyanobacteria. This agrees with what was observed, in laboratory conditions by Sivonen (1990) and Lee et al. (2017) but contradicts what was observed in the field by Sabater et al. (2003). The study carried out by Sabater et al. (2003) pointed out that full light availability was one of the factors that contributed to generate a favorable environmental scenario for benthic cyanobacteria development at the Llobregat river (Catalonia, NE Spain). However, this and other studies carried out in the same river also suggested a primary role of low water flow and nitrogen to phosphorus ratio in the massive growth of *Oscillatoria* mats (Sabater et al., 2003; Vilalta et al., 2003).

The AFDM, AI, and MI were also affected by the different water flows conditions, particularly showing higher values of these parameters under low flow conditions. Ateia et al. (2016) found out that the moderate water flow provided favorable conditions for algal growth and the corresponding biomass accumulation. Nevertheless, the results obtained by Graba et al. (2013) showed that there were no significant differences in AFDM depending on water flow, but this parameter was a selective factor in algal composition. A shift in the algal community composition was also observed in this study, with the proportion of the *Oscillatoria* genus being much higher under low water flow (F1 and F2). Vilalta (2004) described a cyanobacterial growth favored by slow current velocity areas in a field study performed in a Mediterranean river.

Different factors have been described as cyanobacterial blooms promoters. For example, the abundance of cyanobacteria is strongly influenced by nutrient availability, being the abundance maximized under high nutrient concentrations (Vilalta, 2004; Jankowiak et al., 2019). Some of the factors promoting cyanobacterial blooms are consequences of anthropic activities, such as the inefficient treatment of wastewater and the intensification in the use of fertilizers, whereas some other are environmental such as the increment in temperature or the amount of rainfall (Lürling et al., 2018). These effects are of greater importance in rivers affected by water scarcity situations (i.e., Mediterranean streams), where water flow can be extremely reduced, thus increasing nutrient concentration in water. It should be noted that, according to climate change predictions, these water scarcity scenarios will become common (Bangash et al., 2013). It is predicted that the increase in temperature as another consequence of climate change will cause a greater increase in cyanobacterial blooms, since cyanobacteria can have a competitive advantage at elevated temperature (Lürling et al., 2018).

In our study, the highest amount of geosmin in biofilm was detected under low light and low flow conditions, thus confirming the important role of these variables in the production of geosmin by *Oscillatoria* sp. Some studies focused on light effects have found that geosmin produced by *Oscillatoria f. granulata* growing under laboratory conditions increased at lower light Tsuchiya and Matsumoto (1999). Blevins et al. (1995) and Saadoun et al. (2001) also found that reducing the light intensity resulted in the increased production of geosmin in cultures of *Anabaena* sp. The lowest amount of geosmin found at high light conditions in our study could be attributed to the inhibition of the gas vacuole formation in certain cyanobacteria, as was described under intense light situations by Blevins et al. (1995) and Li et al. (2012). Other studies have evaluated the role of water flow on geosmin concentration in the field. In our study, the higher presence of *Oscillatoria* sp. at the low flow conditions suggest a clear relationship with the geosmin concentration, achieving the highest values under low flow treatments. This result highlights the effect of water flow as a critical factor in the geosmin formation, the maximum geosmin production occurs when water flows is approaching basal flow (Vilalta et al., 2003) specially in Mediterranean areas, where water scarcity is a common situation (Casas-Ruiz et al., 2016; Cid et al., 2017; Karaouzas et al., 2018). It is important to highlight that none of the above mentioned studies has previously tested the independent and combined effect of these two factors on geosmin production under controlled conditions in a mesocosms system receiving water directly from the river. The present study is thus the first one filling the lack of information about the joint effect of light and water flow on geosmin formation in freshwater benthic communities.

According to these results, the interaction of both parameters, water flow and light availability, has an important role in the biofilm geosmin formation, by changing the community composition and thus favoring the intracellular geosmin formation. Freshwater systems affected by low light irradiation and low water flow are of special concern because they are the most susceptible to experience geosmin episodes, having an impact on both odor and taste of surface waters. This will have a negative effect on the customers trust, being an important problem for water utilities in those rivers exploited for drinking water purposes.

Under the most currently accepted climate change predictions, it is expected a reduction in river water flows mainly due to a decrease in rainfall events and an increase in air temperature (IPCC, 2018). Thus, the results obtained in this study strongly suggest a probable increase of geosmin episodes. The reduction of water flow favors the appearance of cyanobacteria (such as *Oscillatoria* sp.) which may lead to variations in the nutrient uptake capacity of the biofilm increasing the phosphorus concentration in water. In particular, biofilms capture less phosphorus at lower flows because of (i) biological factors, such as a community shift toward one with a lower nutrient uptake capacity or (ii) by physical factors, such as the limitation in nutrients diffusion due to a greater thickness of the biofilm (Battin et al., 2016). Both situations would lead to an optimum scenario for the formation of geosmin by *Oscillatoria* sp.

The results obtained in this study indicated the potential implications for stream ecosystem management to control geosmin appearance. Likewise, they could be used as an early warning system, establishing that in times of drought, which lead to a decrease in water flow, the situation could be optimal for the appearance and development of geosmin producing cyanobacteria on the banks of rivers.

## Conclusion

Overall, this experimental study shows that both light availability and water flow have a clear effect on the biofilm community and thus on the intracellular geosmin formation. Low light and low water flow empower the *Oscillatoria* genus appearance, providing a favorable situation to start producing geosmin. In order to prevent geosmin episodes in those rivers exploited by drinking water purposes, it is important to further control the water nutrient content, and it will be essential to guarantee the environmental flow regimes to prevent the growth and accumulation of geosmin-producing cyanobacteria.

Considering the Ter river scenario, which is affected by a high presence of dams (one every 800 meters), an important factor to take into account in the management of this system, could be to reduce the presence of these dams, totally or partially, to increase the river flow. As far as possible, nutrient entry points to the system should be identified and reduced, avoiding or minimizing eutrophication in rivers thus preventing geosmin episodes.

## Data Availability Statement

All relevant data is contained within the manuscript.

## Author Contributions

All authors made substantial contributions to the conception or design of the work, or the acquisition, analysis, or interpretation of data for the work.

## Conflict of Interest

The authors declare that the research was conducted in the absence of any commercial or financial relationships that could be construed as a potential conflict of interest.
